# Effects of Oxidative Stress Gene Protein, Expression, and DNA Methylation on Multiple Sclerosis: A Multi‐Omics Mendelian Randomized Study

**DOI:** 10.1002/brb3.70606

**Published:** 2025-05-30

**Authors:** Yang Li, Yushi Wang, Shuning Wang, Hui Zhu

**Affiliations:** ^1^ Department of Neurology The First Hospital of Jilin University Changchun Jilin China

**Keywords:** Mendelian randomization, molecular docking, multi‐omics, multiple sclerosis, oxidative stress

## Abstract

**Background:**

Oxidative stress (OS) is linked to the development of multiple sclerosis (MS), but the causal relationship in terms of genetic pathophysiology remains ambiguous. We employed Mendelian randomization (MR) and colocalization analysis to explore the relationship between OS genes and MS, utilizing an integrative multi‐omics approach.

**Methods:**

We obtained data from a genome‐wide association study (GWAS) of MS from the International Multiple Sclerosis Genetics Consortium (Discovery phase) and the FinnGen study (Replication phase). Mendelian randomization analyses were conducted using summary data to evaluate the association between molecular features of OS‐related genes and MS. Additional colocalization analyses were undertaken to ascertain whether the identified signal pairs shared causal genetic variants.

**Results:**

Integration of multi‐omics data, including mQTL‐eQTL and eQTL‐pQTL, revealed that the *STAT3* gene is associated with MS, supported by Level 1 evidence. The *CR1* gene shows an association with MS risk, evidenced by Level 3 support. Methylation at cg24718015 and cg17833746 in the *STAT3* gene correlates with reduced expression of *STAT3*. At the protein level, high circulating levels of STAT3 are inversely associated with MS risk (OR: 0.43, 95% CI, 0.33–0.54). Elevated levels of TNFRSF1A are also linked with a decreased risk of MS (OR: 0.21; 95% CI, 0.12–0.37), while higher levels of CR1 are positively associated with an increased risk of MS (OR: 1.17; 95% CI, 1.08–1.27).

**Conclusion:**

This study identifies specific OS genes that are associated with MS and enhances our understanding of its pathogenesis.

## Graphical abstract

Mendelian randomization (MR) and colocalization analysis were used to study the relationship between oxidative stress genes and multiple sclerosis by integrating multiple omics, and finally, two significant genes related to STAT3 and CR1 were obtained.

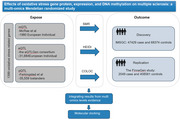

## 1 Background

Multiple sclerosis (MS) is one of the most prevalent nontraumatic, disabling conditions affecting young adults globally. Its incidence is increasing worldwide, significantly impacting individuals' quality of life (Dobson and Giovannoni 2018). MS is characterized by inflammatory demyelinating lesions within the white matter of the central nervous system (CNS), typifying this autoimmune disorder. The etiology and pathogenesis of MS remain incompletely understood. It is hypothesized that oxidative stress (OS) and inflammatory processes contribute to the tissue damage observed in MS (Suneetha and Raja Rajeswari 2016). Specifically, macrophages produce elevated levels of reactive oxygen species (ROS), leading to OS that potentially damages myelin and axons in MS and its animal model, Experimental Autoimmune Encephalomyelitis (EAE) (Offen et al. 2004). Furthermore, ROS can induce cell necrosis or apoptosis by damaging critical cellular components such as proteins, lipids, and nucleic acids (Offen et al. 2004). Although the involvement of OS in MS is well‐recognized, the specific genes implicated in OS and their impact on the disease remain to be elucidated.

Mendelian randomization (MR) is a statistical method that uses genetic variants as instruments to enhance causal inference concerning the associations between exposures and outcomes (J. Chen et al. 2024). This approach leverages genetic variants that are assigned randomly and independently during meiosis, thereby mitigating the effects of confounding factors and reverse causation (Lawlor et al. 2008). With the advent of large‐scale genomic studies and molecular quantitative trait loci (QTL) data, it is now feasible to explore the mechanisms regulating OS‐related genes in MS through analyses of methylation, gene expression, and protein levels. In this study, we applied MR at a multi‐omics level to assess potential links between OS genes and MS.

## 2 Materials and Methods

**FIGURE 1:**
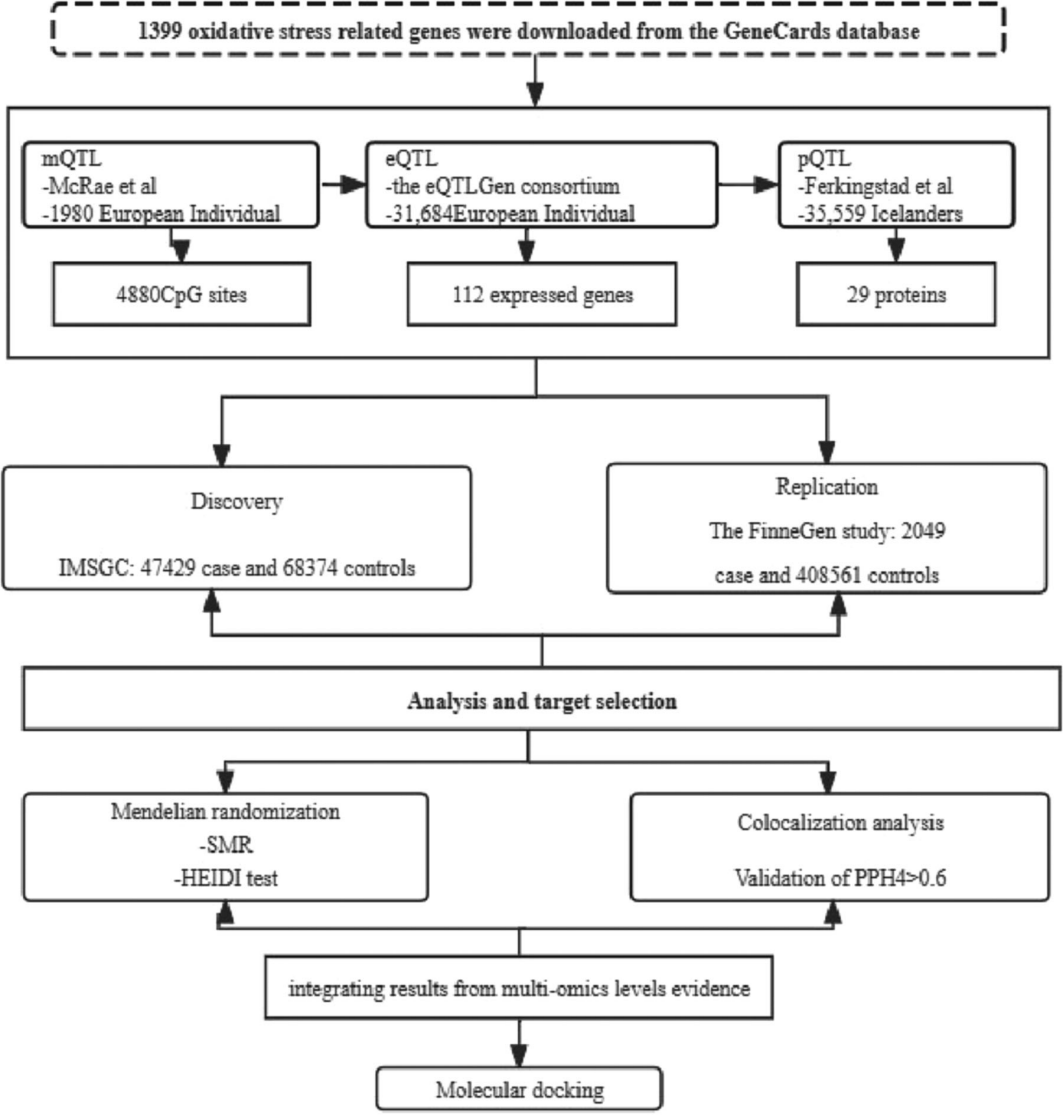
Study design. MS: multiple sclerosis; PPH4, posterior probability of H4; QTL, quantitative trait loci; SMR, summary‐based Mendelian randomization; SNP, single‐nucleotide polymorphisms.

The experimental design is illustrated in Figure 1. Our MR analyses utilized publicly available datasets, including the International Multiple Sclerosis Genetics Consortium (IMSGC) (Patsopoulos et al. 2019), the FinnGen study (Kurki et al. 2023), and other genome‐wide association studies (GWASs) (Table S1). The IMSGC dataset served as the discovery cohort, while the FinnGen R10 cohort was used for validation. We extracted instrumental variables for OS genes from data on methylation, gene expression, and protein abundance. Separate MR analyses were conducted on MS at these different biological levels. To bolster our causal inferences, we also performed a colocalization analysis. The results were further corroborated using data from the FinnGen study. By integrating MR analysis data from the three aforementioned levels, we identified several candidate genes with potential causal associations. No overlap in samples between the exposure and outcome groups was noted.

### 2.1 Data Sources of Exposure

Integrated multi‐omics data can elucidate potential molecular networks implicated in OS. Associations of SNP‐CpGs in blood were obtained from methylation QTL (mQTL) data in 1980 European ancestry by McRae et al., using Illumina HumanMethylation450 arrays (J. Chen et al. 2024; McRae et al. 2018; Y. Wu et al. 2018). Additionally, the expression quantitative trait loci (eQTL) dataset, comprising data from 31,684 individuals, was sourced from the eQTLGen consortium (J. Chen et al. 2024; Võsa et al. 2021). We also incorporated summary statistics for genetic associations with circulating protein levels from a protein QTL (pQTL) study involving 35,559 Icelanders conducted by Ferkingstad et al. (J. Chen et al. 2024, Ferkingstad et al. 2021).

We accessed the GeneCards database to download data on 1399 genes associated with OS, selecting those with a correlation score of ≥ 7, indicative of the top 10% OS‐related genes, for further analysis (Table S2) (H. Wang et al. 2023; Qiu et al. 2020; Fan et al. 2022; Sun et al. 2022). These genes were then cross‐referenced within our QTL datasets to identify OS‐related genes, resulting in the identification of 285 methylation‐related genes, 112 expressed genes, and 29 protein genes, using established thresholds for significance (*p* < 5 × 10^−8^).

### 2.2 MS Outcome Datasets

Data pertaining to MS were obtained from the IMSGC and the FinnGen study. The IMSGC dataset included 47,429 MS cases and 68,374 controls, all of European descent. We also utilized the 10th release of the FinnGen dataset (FinnGen R10), which encompasses the most current sequencing data, clinical information, and genetic analyses, including 2409 cases and 408,561 controls. The IMSGC dataset was employed during the discovery phase of our study, while the FinnGen dataset facilitated the replication phase. Importantly, there was no sample overlap between the two datasets.

### 2.3 Summary‐Data‐Based MR Analysis

We adopted summary‐data‐based MR (SMR) to investigate the relationship between methylation, expression, and protein abundance of genes and MS (Z. Zhu et al. 2016). Cis‐QTLs with the strongest associations from previous studies were selected, excluding SNPs with significant allele frequency differences (J. Chen et al. 2024). The heterogeneity in dependent instruments test (HEIDI) was applied to discern pleiotropy from linkage, with a P‐HEIDI value < 0.01 suggesting a likely pleiotropic effect, leading to exclusion from the analysis (J. Chen et al. 2024). The SMR and HEIDI tests were conducted using SMR version 1.3.1. Furthermore, the Benjamini–Hochberg method was employed to adjust the *p* values, thereby controlling the false discovery rate (FDR) at *α* = 0.05 (J. Chen et al. 2024). We considered associations with adjusted *p* < 0.05 and P‐HEIDI > 0.01 for subsequent colocalization analysis (J. Chen et al. 2024).

### 2.4 Colocalization Analysis

For our colocalization analysis, we employed the coloc package in R version 4.3.1 to investigate potential common causal variants between MS and mQTL, eQTL, and pQTL associated with OS. We conducted the analyses using SNPs that were harmonized utilizing the TwoSampleMR package, adopting default prior probabilities: *p*1 = 1E−4, *p*2 = 1E−4, *p*12 = 1E−5. These probabilities represent the likelihood that a SNP within the tested region significantly correlates with gene expression, MS risk, or both, respectively. The analysis delineated five distinct posterior probabilities that align with the following hypotheses: (H0) no genetic association in the region with SNPs; (H1) only one feature genetically associated with SNPs; (H2) only the second feature genetically associated with SNPs; (H3) both features associated with SNPs, albeit via different dependent variables; (H4) both features associated with SNPs under a shared dependent variable (J. Chen et al. 2024). The designated windows for colocalization regions in pQTL‐GWAS (Yoshiji et al. 2023), eQTL‐GWAS (GTEx Consortium, Laboratory et al. 2017), and mQTL‐GWAS (Morrow et al. 2018) were set at ±1000, ±1000, and ±500 kb, respectively, as informed by prior research. A posterior probability greater than 0.80 for H4 (PPH4) is considered significant support for colocalization.

### 2.5 Integration of Multi‐Omics Evidence

To comprehensively understand the relationship between OS‐related genes and MS across different molecular layers, we integrated data from three gene regulation dimensions. Recognizing that proteins represent the final products of gene expression pathways, we prioritized protein‐level evidence to establish causality. Consequently, we classified causal candidate genes into three tiers based on their associations with MS (J. Chen et al. 2024): Tier 1 genes associated with MS at the protein abundance level (adjusted *p* < 0.05, PPH4 > 0.8), and also at both methylation and expression levels (adjusted *p* < 0.05 for each); Tier 2 genes associated with MS at the protein abundance level (adjusted *p* < 0.05, PPH4 > 0.8), and either at the methylation or expression level (adjusted *p* < 0.05 for each); Tier 3 genes associated with MS at the protein abundance level (adjusted *p* < 0.05, PPH4 > 0.8), and either at the methylation level (nominal *p* < 0.05) or expression level (nominal *p* < 0.05) (J. Chen et al. 2024). Additionally, to further substantiate the credibility of our findings, MR analyses were performed to explore causal relationships between mQTL and eQTL, as well as between eQTL and pQTL.

### 2.6 Molecular Docking

In order to evaluate the binding energy and interaction modes between prospective drugs and their target genes, we conducted molecular docking at the molecular level. This analysis aids in better understanding the drug‐target interactions and the potential efficacy of target genes. We sourced the structural data for drug candidates from the PubChem Compound Database and obtained the corresponding gene protein structure data from the Protein Data Bank. Subsequently, we utilized the computational protein‐ligand docking software, AutoDock Vina version 1.5.6, to perform molecular docking on six drug candidates and the proteins encoded by the relevant target genes.

## 3 Results

### 3.1 OS Gene Methylation and MS

The results demonstrating the causal effects of OS gene methylation on MS are presented in Table 1. After eliminating associations with a P‐HEIDI value less than 0.01, a total of 4,880 CpG sites around 285 distinct genes met the criteria for marginal significance (*p* < 0.05), as shown in Table S3. Subsequent adjustments for multiple testing revealed 930 CpG sites proximal to 53 distinct genes, as detailed in Table 1. Notably, seven CpG sites near three unique genes exhibited strong colocalization evidence (PH4 > 0.80), specifically *EIF4EBP1* (cg19755435, cg02713832, cg20008734) and *BACH2* (cg18477569). Furthermore, the associations of cg19755435 and cg20008734 near *EIF4EBP1*, along with cg18477569 near *BACH2*, were replicated in the FinnGen study (Table S4).

**TABLE 1:** Associations of genetically predicted OS gene methylation with MS in Mendelian randomization analysis.

Gene	probeID	*p* value	*p* value after FDR adjustment	OR (95% CI)
ABCA1	cg14313833	2.27E‐04	8.61E‐03	1.17 (1.08, 1.27)
AGER	cg05436760	2.83E‐09	2.67E‐07	10.09 (4.71, 21.64)
BACH2	cg25204543	7.51E‐09	6.64E‐07	1.46 (1.28, 1.66)
	cg18477569	8.39E‐09	7.31E‐07	1.11 (1.07, 1.14)
BAD	cg25069102	3.29E‐04	0.01	0.76 (0.65, 0.88)
BCL2L11	cg04202892	2.88E‐04	0.01	0.93 (0.89, 0.97)
	cg18646521	4.28E‐04	0.01	0.85 (0.78, 0.93)
CACNA1S	cg24731111	2.60E‐05	1.22E‐03	0.6 (0.48, 0.76)
CD40	cg11841529	6.10E‐05	2.64E‐03	1.4 (1.19, 1.64)
	cg01943874	6.26E‐05	2.70E‐03	1.4 (1.19, 1.65)
	cg17929951	2.24E‐04	8.55E‐03	1.63 (1.26, 2.11)
CDK4	cg03829839	7.24E‐08	5.30E‐06	0.72 (0.63, 0.81)
	cg19542346	2.91E‐05	1.35E‐03	1.59 (1.28, 1.97)
CRYAA	cg22633722	1.24E‐03	0.03	0.72 (0.59, 0.88)
CXCR4	cg06795425	1.77E‐03	0.05	1.17 (1.06, 1.29)
CYBA	cg19790294	1.04E‐03	0.03	0.74 (0.62, 0.89)
	cg03870138	1.04E‐03	0.03	0.73 (0.61, 0.88)
CYP20A1	cg18942464	1.60E‐03	0.04	0.81 (0.71, 0.92)
CYP21A2	cg14026451	2.06E‐05	9.94E‐04	1.75 (1.35, 2.27)
DDAH2	cg11261908	9.49E‐10	9.52E‐08	21.61 (8.07, 57.86)
	cg23760103	3.44E‐09	3.19E‐07	26.86 (9.02, 80.03)
	cg17983217	1.19E‐07	8.36E‐06	0.57 (0.46, 0.7)
	cg00124375	7.14E‐07	4.43E‐05	0.56 (0.45, 0.71)
DGKQ	cg05667917	2.73E‐04	0.01	1.07 (1.03, 1.1)
	cg05178683	1.61E‐03	0.04	0.85 (0.76, 0.94)

*Note*: Only the top 25 probes sorted by gene name are shown.

### 3.2 OS Gene Expression and MS

**FIGURE 2:**
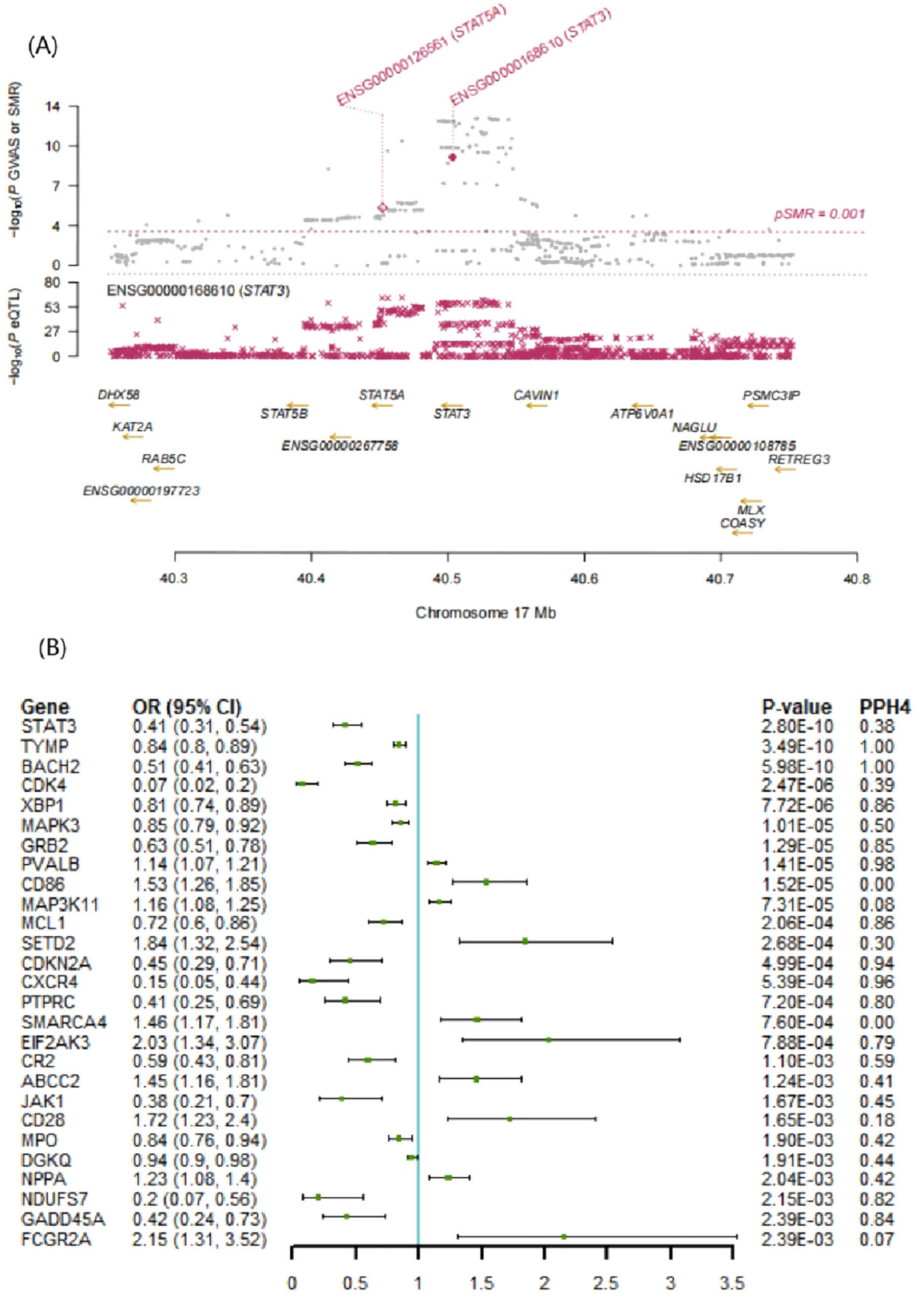
Associations of genetically predicted OS gene expression with MS in Mendelian randomization analysis. (A) Chromosome Loci Map: The top plot displays the *p* values for SNPs from GWAS and SMR tests, with solid diamonds indicating SNPs that pass the HEIDI test. The middle plot illustrates the eQTL results for *STAT3*. The bottom plot indicates gene locations, with probes meeting the SMR threshold highlighted in maroon. (B) Forest Plot. CI, confidence interval; eQTL, expression quantitative trait loci; GWAS, genome‐wide association studies; HEIDI, heterogeneity in dependent instrument; OR, odds ratio; PPH4, posterior probability of H4; pSMR, SMR method *p* value; SMR, summary Mendelian randomization.

Figure 2 illustrates the causal effects of OS gene expression on MS. An analysis revealed that 112 relevant genes were significantly correlated with MS (*p* < 0.05), as detailed in Table S5. Following corrections for multiple tests and colocalization analysis, several genes demonstrated significant associations: *TYMP* (OR: 0.84; 95% CI, 0.80–0.89; PPH4 = 1.00), BACH2 (OR: 0.51; 95% CI, 0.41–0.63; PPH4 = 1.00), *CXCR4* (OR: 0.15; 95% CI, 0.05–0.44; PPH4 = 0.96), *CDKN2A* (OR: 0.45; 95% CI, 0.29–0.71; PPH4 = 0.94), *MCL1* (OR: 0.72; 95% CI, 0.60–0.86; PPH4 = 0.86), *XBP1* (OR: 0.81; 95% CI, 0.74–0.89; PPH4 = 0.86) *GRB2* (OR: 0.63; 95% CI, 0.51–0.78; PPH4 = 0.85), *GADD45A* (OR: 0.42; 95% CI, 0.24–0.73; PPH4 = 0.84) *NDUFS7* (OR: 0.20; 95% CI, 0.07–0.56; PPH4 = 0.82), *PTPRC* (OR: 0.41; 95% CI, 0.25–0.69; PPH4 = 0.80). Conversely, genetically predicted higher levels of *PVALB* expression were positively associated with MS risk (OR: 1.14; 95% CI, 1.07–1.21; PPH4 = 0.98). The associations for *BACH2*, *CXCR4*, *XBP1*, and *NDUFS7* were validated in the FinnGen dataset (Table S6).

### 3.3 OS Proteins and MS

A total of 29 OS proteins were associated with MS risk, all showing significance at *p* < 0.05 (Tables S7 and S8). Following correction for multiple testing, genetically predicted higher levels of STAT3 (OR: 0.43; 95% CI, 0.33–0.54), TNFRSF1A (OR: 0.21; 95% CI, 0.12–0.37), MAPK3 (OR: 0.71; 95% CI, 0.61–0.83), and TLR3 (OR: 0.92; 95% CI, 0.87–0.97) were negatively associated with MS risk. Conversely, higher levels of CR1 (OR: 1.17; 95% CI, 1.08–1.27) and PARP1 (OR: 4.10; 95% CI, 1.75–9.59) were positively associated with MS risk (Table S7). Evidence of colocalization was found for TNFRSF1A (PPH4 = 1.00), STAT3 (PPH4 = 0.98), and CR1 (PPH4 = 0.83) with MS (Table 2).

**TABLE 2:** Associations of genetically predicted OS gene‐encoded proteins with MS in Mendelian randomization analysis.

Gene	*p* value	*p* value after FDR adjustment	OR (95% CI)	PPH4
STAT3	6.03E‐12	4.32E‐10	0.43 (0.33, 0.54)	0.98
TNFRSF1A	7.22E‐08	2.96E‐06	0.21 (0.12, 0.37)	1.00
MAPK3	1.26E‐05	4.51E‐04	0.71 (0.61, 0.83)	0.50
CR1	1.85E‐04	0.01	1.17 (1.08, 1.27)	0.83
PARP1	1.15E‐03	0.03	4.1 (1.75, 9.59)	0.58
TLR3	1.87E‐03	0.04	0.92 (0.87, 0.97)	0.17

### 3.4 Integrating Evidence From Multi‐Omics Levels

After integrating multi‐omics evidence, we identified that the gene *STAT3* is linked to MS with level 1 multi‐omics evidence (Table 3). Examination of target gene‐MS relationships in the FinnGen dataset (replicates) indicated that while not all correlations reached statistical significance, the direction of most correlations remained consistent with our findings (Table S9). MR analysis also revealed a positive correlation between the expression of the identified gene *STAT3* and the corresponding protein levels (Table S10). Methylation at cg24718015 and cg17833746 in *STAT3* was associated with low expression of *STAT3*, consistently showing a positive effect of cg24718015 and cg17833746 methylation on MS risk.

**TABLE 3:** Tier of genetically predicted methylation, expression, and protein levels of candidate genes with MS in Mendelian randomization analysis.

Gene (Tier)	mQTL			eQTL		pQTL	
	Probe	OR (95% CI)	*p* value	OR (95% CI)	*p* value	OR (95% CI)	*p* value
STAT3 (Tier 1)	cg24718015	1.47 (1.28, 1.69)	7.65E‐08	0.41 (0.31, 0.54)	2.80E‐10	0.43 (0.33, 0.54)	6.03E‐12
	cg17833746	1.56 (1.32, 1.85)	1.87E‐07				
CR1 (Tier 2)	cg21110645	1.08 (1.02, 1.14)	3.82E‐03			1.17 (1.08, 1.27)	1.85E‐04

### 3.5 Candidate Drug Prediction

In this study, we utilized the Comparative Toxicogenomics Database (CTD) to identify potentially effective therapeutic agents, selecting the six most promising chemicals as detailed in Table 4.

**TABLE 4:** Docking results of available proteins with small molecules.

Target	PDB ID	Drug	PubChem ID	Binding energy
STAT3	6NJS	Acetaminophen	1983	−5.2
		Estradiol	5757	−7.1
		Quercetin	53790765	−8.1
		Raloxifene Hydrochloride	54900	−7.2
		Streptozocin	29327	−5.4
		Valproic Acid	3121	−4.2

*Note*: The lower the binding energy, the better the binding effect, and the higher the affinity.

### 3.6 Molecular Docking

**FIGURE 3:**
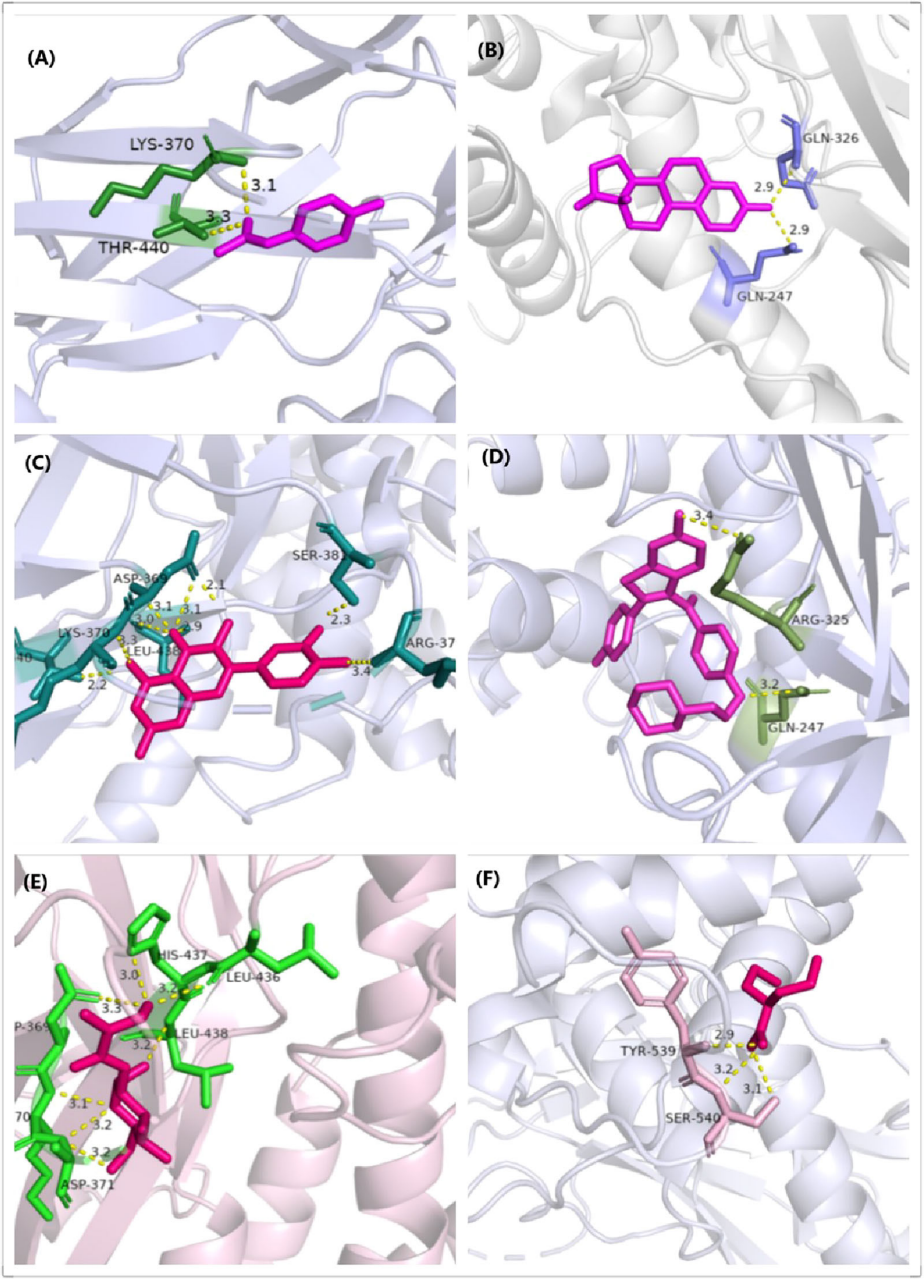
Docking results of available proteins with small molecules. (A) STAT3‐Acetaminophen, (B) STAT3‐Estradiol, (C) STAT3‐Quercetin, (D) STAT3‐Raloxifene Hydrochloride, and (E) STAT3‐Streptozocin, (F) STAT3‐Valproic Acid.

Molecular docking analyses were conducted to assess the binding affinities of six drug candidates to their respective targets, thereby enhancing our understanding of target druggability. Using AutoDock Vina version 1.5.6, we determined the binding sites and characterized the interactions between the drug candidates and the proteins encoded by their respective genes. The binding energies of these interactions were calculated, revealing six significant protein–drug interactions (Table 4) (Cao et al. 2023). Notably, each drug candidate exhibited substantial electrostatic interactions and discernible hydrogen bonds with its protein target (Figure 3). Among these, quercetin displayed the lowest binding energy, at −8.1 kcal/mol, suggesting exceptionally stable binding.

## 4 Discussion

Our study revealed a potential link between the OS‐responsive *STAT3* gene and the risk of MS, supported by multi‐omics evidence. To our knowledge, this is the first investigation to explore the effect of OS on MS pathophysiology using an integrated multi‐omics approach. Prior studies have identified *STAT3* as a critical juncture in numerous oncogenic signaling pathways, playing a pivotal role in modulating the antitumor immune response (Zou et al. 2020) and being regulated by redox changes (Butturini et al. 2020). Extensive genomic research has also associated variants of the *STAT3* gene with MS, particularly noting the SNP (rs744166) within the *STAT3* gene (Jakkula et al. 2010). Microglia‐mediated neuroinflammation is recognized as a characteristic of several neurodegenerative diseases, including MS (Song and Suk 2017). The neuroinflammatory response involves the activation of microglia, which may differentiate into either pro‐inflammatory (M1) or anti‐inflammatory (M2) phenotypes depending on the microenvironmental cues—processes known as classical and alternative activation, respectively (Zhang et al. 2019). M1 microglia contribute to inflammation and neurotoxicity (Colonna and Butovsky 2017), whereas M2 microglia are involved in myelin regeneration and exert neuroprotective and anti‐inflammatory effects (Kuntzel and Bagnard 2022). In MS, there is a predominance of microglia polarized towards an M1‐like phenotype, which is implicated in the damage to myelin sheaths (Kuntzel and Bagnard 2022). Microglia, therefore, play a dual role in the progression of neurodegenerative diseases (Tang and Le 2015).

Functional polarization of microglia could offer new perspectives for understanding how localized CNS inflammation fosters neurodegeneration. Several recent investigations have explored strategies to treat MS by targeting macrophage/microglia polarization. For instance, Weng et al. (2018) demonstrated that lenalidomide could effectively suppress typical CNS inflammatory demyelinating disorders by promoting M2 macrophage polarization. Lenalidomide enhances the prevalence of M2 macrophages and inhibits pro‐inflammatory Th1 and Th17 cells in the CNS, thereby reducing inflammation and demyelination in affected central tissues (Weng et al. 2018). At the cellular level, lenalidomide significantly increases macrophage IL‐10 expression and autocrine secretion, which subsequently phosphorylates STAT3 at tyrosine 705, activating M2‐type macrophage polarization (Weng et al. 2018). Activation of STAT6, AKT, and STAT3 has been shown to shift macrophages from a detrimental M1 phenotype to a beneficial M2 phenotype (G. Wang et al. 2015, Sica and Mantovani 2012). Moreover, given its crucial roles in myeloid cell activation, T‐cell polarization, and cytokine/chemokine production, STAT3 is pathogenically relevant in MS (Qin et al. 2012; E. Zhu et al. 2014).

CD4+ T cells orchestrate the adaptive immune response by secreting cytokines that activate various target cells. Naïve CD4+ T cells can differentiate into at least four subsets: Th1, Th2, Th17, and inducible regulatory T cells. The differentiation of these subsets is triggered by cytokine stimulation, leading to the activation of Stat proteins, which in turn initiate the expression of key transcription factors governing cell fate (Christie and Zhu 2014). Among these, Th17 differentiation is specifically promoted by IL‐6 and TGFβ (Yang et al. 2007), where IL‐6 employs STAT3 signaling. Active STAT3 not only facilitates Th17 differentiation but also enhances the expression of the transcription factor RORγt (Zhou et al. 2007). Conversely, the absence of STAT3 leads to a reduction in Th17 populations and an increase in Treg populations, highlighting the inverse relationship between Th17 and Treg cell differentiation (Yang et al. 2007). The balance between Th17 and Treg cells is crucial, as evidenced by a study that found that a predominance of Th17 cells over Treg cells is a significant contributor to the pathogenesis of MS (B. Wu and Wan 2020). Furthermore, STAT3 activation not only supports the differentiation of Th17 cells but also suppresses both the development and the function of Treg cells. Treg cells mitigate inflammation through the secretion of IL‐10 and TGF‐β, which are essential for maintaining immune tolerance. Excessive STAT3 activity disrupts this balance, leading to heightened immune activity and autoimmune responses. Research has shown that inhibitors of STAT3 can rebalance Th17/Treg dynamics, diminish inflammation in the CNS, and ameliorate the clinical symptoms of EAE, a model for MS (Zhou et al. 2007). In vitro studies have demonstrated that magnolol can bind to STAT3, inhibiting its phosphorylation. This inhibition impedes STAT3's nuclear translocation and transcriptional activity, consequently reducing cytokine levels and Th17 cell differentiation, which ultimately mitigates MS (J. Y. Chen et al. 2023). Most of the aforementioned research focuses on the STAT3 signaling pathways and their cellular impacts, with relatively few studies exploring the association between STAT3 and MS through GWAS.

Our study elucidates the role of OS‐related genes, such as *STAT3*, *CR1*, and *TNFRSF1A*, in the pathogenesis of MS. By integrating multi‐omics data—including methylation, gene expression, and protein levels—we identified *STAT3* as a crucial gene associated with MS risk. This association is supported by MR and colocalization analyses, which enhance our understanding of MS mechanisms, particularly in terms of STAT3's involvement in neuroinflammation and immune regulation. Molecular docking has identified potential drugs targeting STAT3, such as Quercetin and Raloxifene Hydrochloride, opening new therapeutic avenues. This study also underscores the utility of integrating multi‐omics data in researching complex diseases, a method applicable to other neurodegenerative disorders like Alzheimer's and Parkinson's. Our study employs MR and colocalization methods to estimate the causal effects of OS‐related genes, thus reducing bias and enhancing causal inference. By analyzing methylation, expression, and protein levels, we provide a comprehensive view of gene regulation in MS. The use of large‐scale GWAS datasets and consistent results across both discovery (IMSGC) and replication (FinnGen) cohorts strengthens the robustness of our findings. Despite these strengths, our study has several limitations. Firstly, MR cannot account for nongenetic factors, which limits its ability to fully elucidate causal mechanisms. Secondly, our multi‐omics approach does not capture all regulatory mechanisms, such as posttranslational modifications and single‐cell dynamics. Lastly, the findings may lack generalizability due to the datasets' predominantly European ancestry.

Based on our findings, we propose the following directions for future research:

1. Validate the role of *STAT3* in MS using animal models (e.g., EAE) and patient‐derived samples.
2. Optimize and test drug candidates, such as quercetin, in preclinical and clinical trials.
3. Integrate single‐cell transcriptomics and epigenomics to explore regulatory networks in MS.

## 5 Conclusion

Our study explored the potential causal association between methylation, expression, and protein abundance of OS‐related genes and MS, demonstrating that several such genes and their regulation play essential roles in the pathogenesis of MS. Additionally, the study highlighted the medicinal potential of these genes, with molecular docking data indicating their high potential as drug targets.

## Author Contributions

**Yang Li**: writing – original draft, writing – review and editing, methodology, validation. **Yushi Wang**: writing – review and editing, visualization. **Shuning Wang**: writing – review and editing, visualization. **Hui Zhu**: writing – review and editing, writing – original draft, methodology, validation.

## Ethics Statement

This trial was conducted with ethical approval, and we received informed consent from those who participated in the initial study.

## Conflicts of Interest

The authors declare no conflicts of interest.

### Peer Review

The peer review history for this article is available at https://publons.com/publon/10.1002/brb3.70606

## Supporting information



Supplementary Materials.

## Data Availability

The dataset supporting the conclusions of this article is included within the article and its additional file (Supporting Information).
